# Clinical Significance of Serum Zinc Levels in Cerebral Ischemia

**DOI:** 10.4061/2010/245715

**Published:** 2011-02-14

**Authors:** Archit Bhatt, Muhammad U. Farooq, Sailaja Enduri, Clement Pillainayagam, Bharath Naravetla, Anmar Razak, Adnan Safdar, Syed Hussain, Mounzer Kassab, Arshad Majid

**Affiliations:** Division of Cerebrovascular Diseases and Sparrow Hospital William and Claire Dart Stroke Center, A-217 Clinical Center, Department of Neurology and Ophthalmology, Michigan State University, East Lansing, MI 48824, USA

## Abstract

*Background*. Zinc mediates several vital physiological, enzymatic and cellular functions. The association between serum zinc and stroke outcome has not been previously evaluated. *Methods*. This single center retrospective study was conducted on consecutive stroke (*n* = 158) and TIA (*n* = 74) patients. We sought to determine whether serum zinc concentrations in patients with acute ischemic strokes were associated with stroke severity and poor functional status at discharge, respectively. *Results*. Overall, out of the 224 patients analyzed (mean age 67 years), 35.7% patients had low zinc levels (65 mcg/dL). Patients with stroke (*n* = 152) were more likely to have low zinc levels (OR = 2.62, CI 1.92–3.57, *P* < .003) compared to patients with TIA (*n* = 72). For patients with stroke (*n* = 152), multivariate analysis showed that low serum zinc levels (OR 2.82, CI 1.35–5.91, *P* = .035) and strokes with admission severe strokes (NIHSS > 8) (OR 2.68, CI 1.1–6.5, *P* = .03) were independently associated with poor functional status (MRS > 3) at discharge from the hospital. *Conclusion*. Low serum zinc concentrations are associated with more severe strokes on admission and poor functional status at discharge.

## 1. Introduction

Zinc is one of the most abundant trace elements in the body. Second only to iron, it mediates several vital physiological functions and is essential for maintaining a healthy immune system and meeting metabolic demands. Preclinical studies have extensively investigated the role of zinc in cerebral ischemia [1]. However, whether zinc exerts neuroprotective or neurotoxic effects during cerebral ischemia is still unclear. 

No clinical study has investigated an association of serum zinc levels in stroke and transient ischemic attack (TIA). Mean zinc levels reported in various healthy cohorts range from 70 ± 32 mcg/dL [2] to 105.2 mcg/dL [3, 4]. These levels tend to decrease with age but in diabetics appear to remain constant throughout life. No significant gender differences exist in plasma zinc concentrations in healthy or diabetic individuals [2]. We determined zinc levels in patients with TIA and ischemic stroke and examined whether low zinc levels (≤65 mcg/dL) are associated with higher stroke severity (NIHSS > 8) and poor functional status at discharge (MRS > 3). 

## 2. Methods

Consecutive patients admitted with a diagnosis of stroke or TIA between Oct. 2007 and Oct. 2008 were included in this study. Stroke was defined as evidence of high MRI signal on diffusion weighted imaging (DWI). TIA was defined as focal neurological symptoms lasting for less than 24 hours without evidence of stroke on DWI imaging. All zinc levels were obtained within 24 hours of admission. Blood samples were analyzed by atomic absorption spectroscopy; the reference range was 65–150 mcg/dL. 

We retrospectively reviewed charts and extracted demographic data, clinical data, and zinc levels. Based on previous studies, low zinc levels were defined as ≤65 mcg/dL [2–4]. In our population, median zinc levels were 66 mcg/dL. Therefore, we divided zinc levels into two groups: low levels (≤65 mcg/dL) and normal levels (>65 mcg/dL or 66 mcg/dL and higher). A severe stroke was defined as (NIHSS > 8). For patients with stroke, poor functional status at discharge was defined as (MRS > 3). NIHSS and modified Rankin Scales were determined by a neurologist on all patients on admission (within 1 hour) and on discharge. 

SPSS 11.0 was used for data analysis. We also performed univariate analysis to determine if an association exists between low zinc levels and factors such as age, sex, atrial fibrillation, congestive heart failure, coronary artery disease, hypertension, and stroke severity (NIHSS > 8). Pearson's correlation was used to test the general trend of zinc levels with age. Categorical variables were analyzed using chi-square and continuous variables were analyzed using Students' *t*-test. *T* test was used after confirming a normally distributed data using Q-Q plot. Multivariate logistic regression analysis was done to evaluate factors (age, sex, atrial fibrillation, congestive heart faliure, coronary artery disease, diabetes, hypertension, and stroke severity) on poor functional discharge status (MRS > 3 versus MRS ≤ 3). Statistical significance was calculated for 95% CI and *P* < .05. 

## 3. Results

We identified 232 patients with ischemic stroke or TIA—158 patients (68.1%) had the diagnosis of ischemic stroke and 74 patients (31.9%) patients had the diagnosis of TIA. We excluded two patients who were on zinc supplements and 6 patients who had medical care withdrawn or died during the hospitalization. We analyzed a total of 224 patients (152 with stroke and 72 with TIA). Overall, the median zinc levels were 66 mcg/dL. Mean zinc levels (mean ± SD) were 71 ± 26 mcg/dL. In patients with stroke, mean NIHSS was (8 ± 6.6) and median NIHSS was 8. The demographic factors were similar in patients with TIA and stroke (Table 1).

Overall, 35.7% of patients had low zinc levels (≤65 mcg/dL). Patients with stroke were more likely to have low zinc levels (OR = 2.62, CI 1.92–3.57, *P* < .003) compared to patients with TIA. In patients with stroke, 57.62% patients had low zinc levels, and 22% of TIA patients had low zinc levels. We also found a significant difference (*P* = .032) in the mean zinc levels in patients with stroke (68 ± 29 mcg/dL) compared to patients with TIA (76 ± 28 mcg/dL). 

In a univariate data analysis of stroke patients, we found that low zinc levels (OR 2.88, CI 1.31–6.32, chi-square *P* = .009) were associated with more severe strokes. Thirty percent (27 out of 89) of patients with less severe strokes (NIHSS ≤ 8) and 55.5% patients (35 out of 63) with more severe strokes (NIHSS > 8) had low zinc levels. Mean zinc levels in patients with severe strokes (NIHSS > 8) were significantly lower (62 ± 23 mcg/dL) (*P* = .04) compared to strokes with (NIHSS ≤ 8), (73 ± 15 mcg/dL). There were no differences in demographic characteristics in patients with low zinc levels versus normal zinc levels (Table 2).

For patients with stroke (*n* = 152), we conducted multivariate logistic regression analysis to adjust for the effect of factors such as age, sex, HTN, DM, admission NIHSS, atrial fibrillation, and congestive heart failure on poor functional status (MRS > 3) at discharge. Our adjusted analysis showed that only low serum zinc levels (OR 2.82, CI 1.35–5.91, *P* = .035) and NIHSS > 8 on admission (OR 2.68, CI 1.1–6.5, *P* = .03) are independently associated with poor functional status (MRS > 3) at discharge. Other factors did not significantly predict poor functional discharge status. Seventy-two percent (39 out of 53) of patients with poor functional discharge, but only 48.4% (48 out of 99) of patients with good functional discharge status, had low zinc levels (chi-square *P* value =.008). Additionally, we found that mean zinc levels in the patients with poor functional status at discharge (MRS > 3) were 63 ± .29 mcg/dL and were significantly lower compared to patients with MRS ≤ 3) at discharge (73 ± .29 mcg/dL) (*P* = .045). 

## 4. Discussion

Our study is the first to show an association between low zinc levels and cerebral ischemia. Our study showed that lower zinc levels (≤65 mcg/dL) are associated with severe strokes (NIHSS > 8) and independently associated with poor functional status at discharge (MRS > 3). 

Preclinical studies have extensively evaluated the role of zinc in cerebral ischemia and it is still unclear whether zinc is neurotoxic, neuroprotective, or both [1]. Several of these studies evaluating neurotoxic effects, have demonstrated that elevated intracellular zinc levels [5, 6] and zinc supplementation during cerebral ischemia may enhance neuronal death [5]. Zinc inhibition achieved through chelation [7–9] may reduce zinc-mediated neurotoxicity. Conversely, data supporting the neuroprotective effects of zinc have been published. Animal-based studies have shown that zinc supplementation reduces infarct volume [10–12], while zinc chelation [13] is neurotoxic. Also, zinc is an essential and integral part of some critical enzyme systems like SOD (super-oxide dismutase) [14] and matrix metalloproteinases [15], which may enhance or contribute to tissue injury in ischemia. 

Clinical studies have shown an association between low zinc levels (≤70 mcg/dL) and higher mortality in elderly patients with pneumonia [16] and higher incidence of opportunistic infections in AIDS patients with zinc levels ≤65 mcg/dL [17]. It has also been shown that zinc levels tend to fall in myocardial infarction, but the effect on outcomes after myocardial infarction has not been determined [18]. Zinc supplementation has been shown to improve cancer survival [19] and promote wound healing [4]. Also, the value of zinc supplementation [20] and zinc chelation [21] in stroke patients is safe; however, their role in efficacy is unclear at this time and may warrant further study. A study also suggested that low zinc levels may be in fact a risk factor for stroke [22]. A prevention trial, found that zinc supplementation in a Chinese cohort of 29584 persons did not change the rate of stroke death significantly (RR 0.99 (0.84–1.18) [23]. 

Our study does have limitations. Firstly, it is a single-center study devoid of any normal controls and long-term outcomes. In addition, prestroke functional status of patients was unavailable. This may be important as patients with poor nutritional status may have a lower zinc levels and suboptimal functional status. However, our analysis was adjusted for admission NIHSS and discharge MRS, which suggests that an association of low zinc levels and physical disability exists in stroke patients. Whether this is related to stroke remains to be determined. Also, zinc levels are affected by diurnal variations [24], postprandial and preprandial variations, and stress. However, our study, for the first time, indicates that lower serum zinc levels may be associated with stroke severity and extent of disability at discharge. Lower zinc levels were associated with more severe strokes on admission and with poor functional status at discharge. As this is an exploratory study, a large independent epidemiological prospective study is necessary to delineate the importance of serum zinc levels in patients with ischemic stroke and to investigate whether low zinc levels are associated poor long-term outcomes. 

## Figures and Tables

**Table 1:** Demographic characteristics (*N* = 224).

	All patients (*n* = 224)	Ischemic stroke (*N* = 152)	TIA (*N* = 72)
Mean Age yrs (mean ± SD)	67.4 ± 16	66.2 ± 18	69.1 ± 20
Males (*N*)	112	75	37
Atrial fibrillation (*N*)	67	60	7
Congestive heart failures (*N*)	13	11	2
Coronary artery disease (*N*)	72	51	21
Diabetes mellitus (*N*)	75	56	19
Hypertension (*N*)	126	80	46
Smoking (*N*)	53	43	10
Dyslipidemia (*N*)	101	77	24
Admission NIHSS stroke (mean ± SD)	NA	8 ± 6.6	NA
Zinc levels (mean ± SD)	71 ± 26 mcg/dL	68 ± 29 mcg/dL	76 ± 18 mcg/dL

**Table 2:** Characteristics of patients with Ischemic Stroke and association with Low Zinc levels (≤65 mcg/dL).

	Total *N* = 152	Zinc level *N* = 88 (≤65 mcg/dL)	Zinc level *N* = 64 (>65 mcg/dL)	*P* value^#^
Mean age yrs (mean ± SD)*	66.2 ± 18	67.2 ± 19	65.2 ± 16	*P* = .62
Males (*N*)	75	44	31	*P* = .15
Atrial fibrillation (*N*)	60	33	27	*P* = .32
Congestive heart failures (*N*)	11	7	4	*P* = .11
Coronary artery disease (*N*)	51	30	21	*P* = .18
Diabetes mellitus (*N*)	56	36	20	*P* = .10
Hypertension (*N*)	80	55	35	*P* = .15
Smoking (*N*)	43	25	18	*P* = .40
Dyslipidemia (*N*)	77	41	36	*P* = .48
Admission (*N*) NIHSS stroke >8	63	35	28	*P* = .32

^#^All other *P* values calculated by presence or absence of demographic variable versus percentage of patients with low zinc levels (≤65 mcg/dL). *T* test used for continuous variables and *z* test used to test differences in proportions.
